# Next‐generation field courses: Integrating Open Science and online learning

**DOI:** 10.1002/ece3.7009

**Published:** 2020-11-20

**Authors:** Sonya R. Geange, Jonathan von Oppen, Tanya Strydom, Mickey Boakye, Tasha‐Leigh J. Gauthier, Ragnhild Gya, Aud H. Halbritter, Laura H. Jessup, Sara L. Middleton, Jocelyn Navarro, Maria Elisa Pierfederici, Julia Chacón‐Labella, Sehoya Cotner, William Farfan‐Rios, Brian S. Maitner, Sean T. Michaletz, Richard J. Telford, Brian J. Enquist, Vigdis Vandvik

**Affiliations:** ^1^ Department of Biological Sciences University of Bergen Bergen Norway; ^2^ Bjerknes Center for Climate Research Bergen Norway; ^3^ Section for Ecoinformatics & Biodiversity Department of Biology Aarhus University Aarhus C Denmark; ^4^ Center for Biodiversity Dynamics in a Changing World Department of Biology Aarhus University Aarhus C Denmark; ^5^ Department of Ecology Environment and Plant Sciences Stockholm University Stockholm Sweden; ^6^ Department of Environmental Science Policy and Management University of California Berkeley CA USA; ^7^ Department of Geography University of Waterloo Waterloo ON Canada; ^8^ Department of Forestry and Natural Resources Purdue University West Lafayette IN USA; ^9^ Department of Ecological Science and Engineering Purdue University West Lafayette IN USA; ^10^ Department of Zoology University of Oxford Oxford UK; ^11^ Department of Ecology and Evolutionary Biology University of Arizona Tucson AZ USA; ^12^ Faculty of Environmental Sciences and Natural Resource Management Norwegian University of Life Sciences Ås Norway; ^13^ Department of Biology Teaching and Learning University of Minnesota Minneapolis MN USA; ^14^ Living Earth Collaborative Washington University St Louis MO USA; ^15^ Center for Conservation and Sustainable Development Missouri Botanical Garden St Louis MO USA; ^16^ Department of Botany and Biodiversity Research Centre University of British Columbia Vancouver BC Canada

**Keywords:** career development, early career researchers, FAIR principles, higher education, pedagogy, reproducible research

## Abstract

As Open Science practices become more commonplace, there is a need for the next generation of scientists to be well versed in these aspects of scientific research. Yet, many training opportunities for early career researchers (ECRs) could better emphasize or integrate Open Science elements. Field courses provide opportunities for ECRs to apply theoretical knowledge, practice new methodological approaches, and gain an appreciation for the challenges of real‐life research, and could provide an excellent platform for integrating training in Open Science practices. Our recent experience, as primarily ECRs engaged in a field course interrupted by COVID‐19, led us to reflect on the potential to enhance learning outcomes in field courses by integrating Open Science practices and online learning components. Specifically, we highlight the opportunity for field courses to align teaching activities with the recent developments and trends in how we conduct research, including training in: publishing registered reports, collecting data using standardized methods, adopting high‐quality data documentation, managing data through reproducible workflows, and sharing and publishing data through appropriate channels. We also discuss how field courses can use online tools to optimize time in the field, develop open access resources, and cultivate collaborations. By integrating these elements, we suggest that the next generation of field courses will offer excellent arenas for participants to adopt Open Science practices.

## Graphical abstract

Open Science methodology is becoming an increasingly important goal of scientific research. Yet, our higher education system does not necessarily reflect these developments, even though such methods are beneficial for the advancement of early career researchers. Here we highlight how field courses can be a valuable resource for early career researchers and our learning of best practice in Open Science methodology, and how online workflows can support this process.

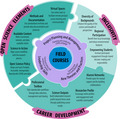

## 1 INTRODUCTION

### 1.1 Benefits of field courses

Field courses are off‐campus university courses in a natural setting and usually follow a collaborative, project‐based structure (Beltran et al., 2020; Durrant & Hartman, 2015; Fleischner et al., 2017). For field course participants, including early career researchers (ECRs—those conducting research as part of a degree program, e.g., Master's or PhD, up to 5 years post PhD), field courses provide a platform for problem‐based learning (Barrows, 1986; Biggs & Tang, 2011) where they have the opportunity to not only apply theoretical knowledge, but to practice relevant methods and tackle the challenges of real‐life research (D'Amato & Krasny, 2011; Durrant & Hartman, 2015; Hole, 2018; Kervinen et al., 2018). In particular, field work experience is crucial to mastering the skill of developing the “research conceptual framework” that is the backbone of the scientific process. With this in mind, field‐based learning experiences can improve learning outcomes by offering opportunities for practical application of skills (Klingberg, 2012) and theory in novel systems and locations (Goodenough et al., 2014). The hands‐on approach of field courses also allows ECRs to develop key “soft skills” such as time management and collaboration with colleagues, especially those from different backgrounds (e.g., educational, cultural, geographic, Leon‐Beck & Dodick, 2012). Field courses not only provide a low‐risk way for ECRs to experience the challenges and opportunities of field‐based research; they also present opportunities for broader professional development including problem‐solving, initiative, confidence, flexibility, intra‐ and interpersonal skills, and leadership capacity (Beltran et al., 2020; Durrant & Hartman, 2015; Fleischner et al., 2017; Kricsfalusy et al., 2018; Peasland et al., 2019). As a result, field course participants may be more adept at problem solving and more confident in a range of working environments. However, while traditional field courses promote real‐world scientific practices, their design may not accurately reflect the cultural shift toward Open Science.

### 1.2 The growth of Open Science and online arenas

Open Science is a global movement which can be broadly summarized as aiming to promote integrity, repeatability, and transparency across all aspects of scientific research including: *open access* to publications, *open data* underlying research, *open source* code and data processing, and increasingly the broader concept of *open reproducible research* (Fecher & Friesike, 2013; Haddaway, 2018; Hampton et al., 2015). FAIR Open Science research practices (Findable, Accessible, Interoperable, Reproducible) (Lannom et al., 2020; Wilkinson et al., 2016) and policies are being increasingly adopted throughout the scientific community, impacting everything from scientific publication via funding agencies to institutional and national research policies (e.g., Coalition‐S, 2020; European Commission, 2018, 2020; Tennant et al., 2019). As such, there is a growing need for ECRs to be well versed in the different aspects and practices of Open Science if they are to be prepared for a future in science (Allen & Mehler, 2019; Farnham et al., 2017). Yet, Open Science practices are not commonly emphasized or integrated in ECR education. Given these practices intersect with many of the key components of field courses (e.g., field campaign design and planning, data collection, data management, report/manuscript writing), field courses could provide an excellent platform for training in Open Science practices. Moreover, as Open Science becomes increasingly integrated with online communities (Luc et al., 2020; Sugimoto et al., 2017; Van Noorden, 2014) and educational practice (Allen & Seaman, 2011; Christensen et al., 2013; Gore, 2014), field courses could make better use of online resources, both to make existing field courses more efficient and to share resources and knowledge among a wider audience.

### 1.3 Taking existing discipline practices as a starting point for incorporating Open Science into field courses

In developing Open Science practices for field courses, ecological and evolutionary disciplines and other field sciences can draw upon existing frameworks for collaborative research, coordinated experiments, data sharing, and data repositories. The importance of developing research projects with reproducibility in mind is exemplified in the recent rise of cooperative research networks (Fraser et al. 2013). Within these research networks, having clearly stated aims and well documented research protocols allows for large quantities of reliable, robust empirical data to be collected and compared across the globe. Examples of these networks within ecology include the International Tundra Experiment (ITEX, Henry & Molau, 1997), which focuses on comparing a specific ecosystem across the globe, while the Nutrient Network (NutNet, Borer et al., 2014) and Soil Temperature initiative (SoilTemp, Lembrechts et al., 2020) facilitate comparisons of abiotic drivers across different ecosystems. Even in instances where researchers are not explicitly working together, ecological sampling follows standardized data collection protocols. Certain protocols focus upon the collection of abiotic or biotic data (e.g., the plant functional trait handbook, Perez‐Harguindeguy et al., 2013), while others outline experimental approaches (e.g., the handbook for standardized field and laboratory methods, Halbritter et al., 2019). Both collaborative projects and standardized protocols have facilitated the development of trait‐based data repositories (e.g., TRY, Kattge et al., 2020), and many of these repositories are increasingly adopting Open Science practices (e.g., Open Traits, Gallagher et al., 2020) Collectively, these approaches encourage data to be available in a standardized format, enabling comparative analysis and evidence synthesis to be conducted (Gallagher et al., 2020; Nakagawa et al., 2020). Lastly, ecological and evolutionary fields have recently started encouraging the use of registered reports (Chambers, 2019), and the publication of citable datasets (Mongeon et al., 2017). Designers of field courses should consider these existing discipline practices as a useful baseline, ensuring courses not only provide relevant ECR training, but in the long term are also able to contribute back to the broader scientific community.

### 1.4 Our field course experience

**Figure 1:**
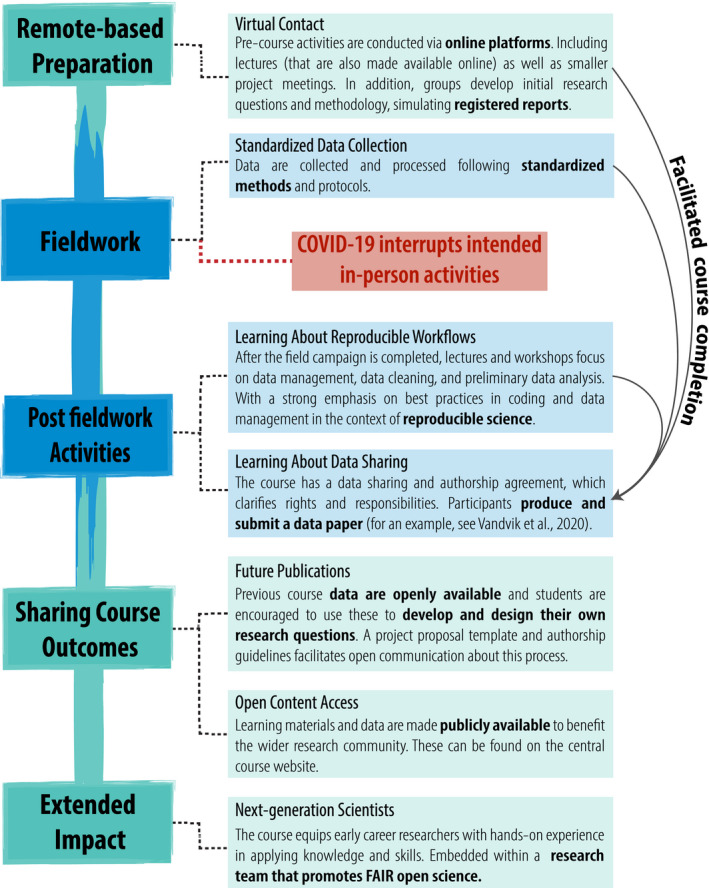
An overview of the general course structure of the 5th Plant Functional Traits Course (PFTC) (remotely based elements in green, in‐person elements are in blue). Here, we highlight how various elements of Open Science and online learning are incorporated throughout the course and how some of these elements allowed the course to run to completion despite disruptions due to COVID‐19 (indicated by the arrows). For more information about these courses, seehttps://plantfunctionaltraitscourses.w.uib.no/

Practical training and field work in ecology has been disrupted by the COVID‐19 pandemic (Barton, 2020; Inouye et al., 2020; Park, 2020), which was also the case with the fifth Plant Functional Trait Course (PFTC) in Peru in March 2020. We were part of this course as students, course instructors, or overall course developers. This course was part of a series that offers ECRs hands‐on training in trait‐based plant ecology in a field course setting. The course covers the theory and methods of key subjects in trait‐based ecology, including plant ecophysiology; community, ecosystem, and climate change ecology; computational biology; and data management and analysis, with an overall emphasis on research reproducibility and Open Science principles. The PFTC format is a mix of remote pre‐ and postfieldwork lectures and workshops, with a two‐week intensive field component (Figure 1). The course activities are centered around collaborative development and execution of field‐based research projects in trait‐based ecology, where the aim is for each group of students to produce and report research‐grade publishable datasets. In the 2020 edition, the two‐week intensive field component was interrupted by the COVID‐19 developments resulting in half the course participants returning to locations around the world, while the remainder were in lockdown in Peru (see Cotner et al., 2020). During this time, components such as online communication platforms and resources, reproducible workflows, and data sharing practices, enabled us to complete our research projects and fulfill the broader educational objectives of the course. Our experience highlighted the usefulness of online communication tools and lectures, along with reproducible Open Science practices (Figure 1), and inspired us to think further about the benefits of integrating these approaches into field course design.

## 2 A NEW APPROACH FOR FIELD COURSES

**Figure 2:**
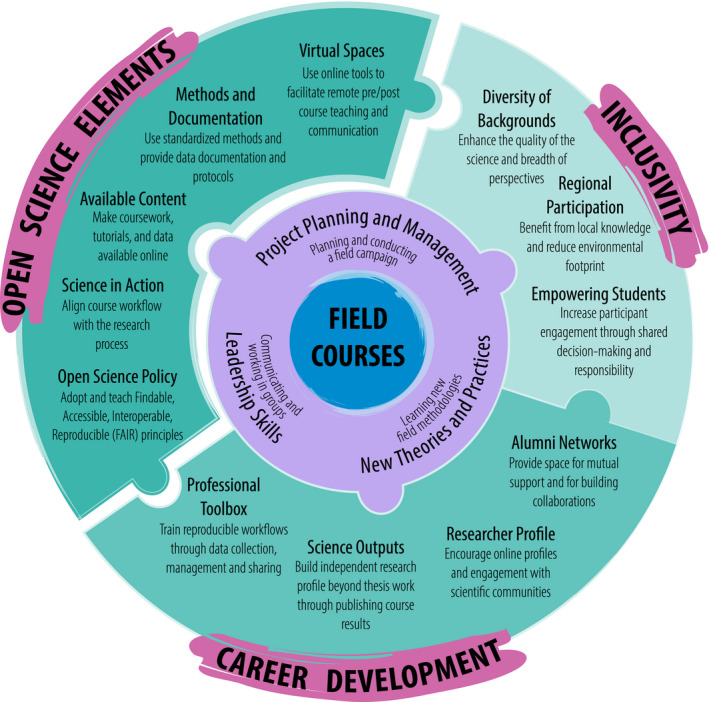
Broadening learning outcomes from field courses through Open Science and online resources. Traditionally, field courses focus on training participants in relevant practical field research methodologies, while also developing their project management and collaborative skills. Here, we illustrate how Open Science research principles could be more explicitly integrated into field course design to enhance broader scientific learning outcomes, while also facilitating early career development and inclusivity in science

Here, we draw together the above points and outline practical recommendations for integrating Open Science practices into field courses (Figure 2) in a way that equips early career researchers with the skills and competences they need to succeed in the future. While our suggestions primarily focus upon graduate courses in ecology and evolution, they are also relevant for other scientific disciplines or project‐based field courses within higher education.

### 2.1 Frame project proposals as registered reports

Field courses often encourage participants to develop research proposals for group work prior to leaving for the field. These proposals (a) are typically based in ecological theory, (b) use common methodology for the discipline, (c) are statistically rigorous, and (d) are often reviewed by course teaching assistants or coordinators. Given this, field course proposals could easily be used to promote Open Science practices, for example by framing them in the context of registered reports, which are becoming increasingly adopted in ecological and evolutionary journals (Chambers, 2019). Registered reports are a relatively new way of publishing scientific work in ecology and evolution, and require authors to submit their proposed research project for peer review prior to the research being conducted, including presenting introductory literature research, and outlining research aims, methods, and proposed analyses. Our suggestion expands on existing practices by explicitly highlighting how this crucial process leads into the publication pathway. Registered report manuscripts and data must be hosted in public repositories, for example, the Open Science Framework (Foster & Deardorff, 2017). To emphasize the importance of project proposals and to facilitate their development, workshops or seminars that explain the links between proposals, registered reports, and publications should be integrated into precourse components of field courses. This could also include hands‐on training in relevant skills, including data management plans.

### 2.2 Align course sampling protocols with discipline standards

Field courses offer a unique chance to teach the importance of Open Science at the start of the data collection pipeline. Most ecological field courses focus primarily on project design, data collection, and/or summarizing the main findings. In comparison, less attention is paid to best practices for creating and managing the data. Field courses should take the opportunity to explicitly specify how projects will adopt standard methodological practices, along with outlining how data and metadata will be collected and stored to be compatible with discipline‐specific data standards and repositories (e.g., Darwin Core, Wieczorek et al., 2012).

### 2.3 Adopt reproducible workflows

We advocate the development of field courses where participants are taught the essentials of reproducible practices for collecting and preparing data. There is often a lack of emphasis on critically important skills for reproducible workflow and data management (see survey responses within Barton, 2020), although this is improving (see Toelch & Ostwald, 2018). Developing reproducible workflows includes: (a) defining the contents of data files; (b) clarifying and defining units, parameters names, and formats; (c) the importance of consistent data organization and file structure; (d) the role of performing quality assurance; and (e) the importance of providing documentation, data dictionaries, and metadata, and making the full data and code openly available (Barton et al., 2010; Borer et al., 2009; Cook et al., 2001; Michener, 2006; Strasser et al., 2018). Working with real datasets when learning these practices can enhance learning outcomes as ECRs will encounter common challenges when handling and cleaning raw data and setting up reproducible code‐based data processing.

Field courses should provide supporting structures for participants to learn, taking into account different participant backgrounds and support needs. For example, field course workflows could include tutorials introducing participants to: script‐based data management and analysis based on open source software; version control systems to track changes; data cleaning and management; code development; and collaborative strategies (Borregaard & Hart, 2016; Vuorre & Curley, 2018). Online platforms can be used as teaching tools to introduce coding and noncoding approaches to explore datasets. Collectively, these approaches facilitate sharing of data and code and are therefore useful not only in field course projects, but also for reproducible research, remotely working with supervisors or collaborators, or when seeking advice, that is, from statisticians or online support groups (for a comprehensive list of approaches and tools, see Alston & Rick, 2020; Hampton et al., 2015). More broadly, these tools promote a culture of transparency and data stewardship, ensuring the integrity and reproducibility of scientific outputs (Powers & Hampton, 2019).

### 2.4 Data documentation and structured datasets as course outputs

The process of creating standardized and reproducible data documentation files and datasets is a learning outcome in itself; however, these resources should also be seen as key course outputs in their own right. Datasets (a) act as valuable resources for designing tutorials on data management and analysis using course‐specific worked examples; (b) can be used as a substitute when scheduled field courses are disrupted—for example, due to bad weather, equipment failure, or unexpected events such as restricted access to field sites, during periods of political unrest, or disease outbreaks (e.g., COVID‐19); and (c) provide the basis for further publication opportunities outside the immediate scope of the individual field course.

### 2.5 Publish course data and data documentation

Field courses can contribute to the broader research community, and provide opportunities for ECRs to gain insights into the publication process, by publishing course data or research outputs. In combining the initial proposals with comprehensive metadata on methodological practices in the field and reproducible data workflows, a strong basis is provided for transforming data documentation files into citable data papers, with benefits for all participants, as well as for making data available for research (see Vandvik et al., 2020). When standard practices and metrics are adopted, field courses can directly contribute data to global data repositories. Furthermore, field courses encouraging ECRs to produce open code and data publications as part of their research workflow will enhance the general availability of reproducible, robust ecological data. This not only benefits the broader scientific community (Lowndes et al., 2017; Parker et al., 2016; Wolkovich et al., 2012), but also increases participants’ hireability (Feng et al., 2020). Beyond data papers, field course participants themselves will benefit from “data impact” indexes, which acknowledge code (Mislan et al., 2016), datasets (Cousijn et al., 2019; Ewers et al., 2019), or contributions to Open Science in general (Mongeon et al., 2017). ECRs who commit to FAIR research practices (Findable, Accessible, Interoperable, Reproducible) (Lannom et al., 2020; Wilkinson et al., 2016) and contribute open data are more likely to have their research included in evidence synthesis research, such as systematic reviews and meta‐analyses (Gurevitch et al., 2001; Haddaway & Verhoeven, 2015). These synthesis approaches may become a more critical component of the Open Science movement in the future (Nakagawa et al., 2020), as they help to identify knowledge gaps, generate new insights, and reduce research waste (Grainger et al., 2020). Under the proposed “data impact” metrics scheme, ECRs would receive additional citations for these contributions. However, until data impact metrics become mainstream, we encourage ECRs to highlight their contributions to Open Science on their CV.

### 2.6 Constructively aligned field courses

In order to realize the full potential of integrating Open Science approaches and best practices into field courses, relevant field course activities and assessment tasks should be constructively aligned with course learning outcomes (Biggs & Tang, 2011). As a first step, key Open Science practices used should be explicitly formulated as part of the intended learning outcomes of the field course (Biggs, 1996), something that is not standard practice currently (see overview in Barton, 2020). Secondly, many of these learning practices (relating to research planning and protocols; data collection, documentation, and management; data sharing and publication) are artifacts that lend themselves to assessment, creating a relatively straight‐forward path to constructively aligning field course learning objectives with assessment tasks and marking guidelines (Biggs & Tang, 2011). Additionally, we argue that the approach we suggest here takes problem‐based learning (Savery, 2015) and “alignment” a step further: by training the ECRs in critical Open Science research skills and assessing their mastery of these skills in the process of conducting real, publishable research, we are essentially immersing or aligning the entire philosophy of field course education within ecological and evolutionary research itself. With this approach, the next generation of field courses will offer excellent arenas for participants to learn best Open Science practices while doing real, publishable research.

## 3 ENHANCING FIELD COURSES WITH ONLINE TOOLS

One of the key strengths of field courses is the “hands‐on” delivery and in‐person engagement (Beltran et al., 2020), and our discussion above illustrates how the incorporation of FAIR Open Science practices and online components can strengthen, add value, and align the key learning outcomes of field courses. The role of online approaches is of particular focus currently, with the ongoing disruptions to in‐person teaching due to COVID‐19 (Barton, 2020; Hines et al., 2020). Not only are online tools complementary to Open Science, but they could prove to beneficial in light of disruptions to field courses (e.g., as outlined in Figure 1). However, there are challenges in shifting ecological learning to online‐only platforms: for instance, learning outcomes traditionally found in field courses are often reduced when shifted to online substitutes (Barton, 2020). Below we focus not on how to move field courses online entirely, but rather highlight how better integration of online resources into existing field course design can be used to prioritize contact time, create online open access resources, and build local and global research networks. In addition, we acknowledge challenges and important considerations in developing online contents.

### 3.1 Using online platforms to prioritize contact time

Field courses can make use of suitable online resources to better optimize the time used in preparation and fieldwork. Online approaches include the following: shifting introductory material and preparation to live, online activities during the precourse phase; using online team meetings to solidify group project frameworks and hypotheses before the field component of the course; and adding data curation and documentation tasks as remote postcourse activities. Adopting these workflows allows for efficient use of time in the field, for example, the collection of more data or shorter field campaigns. In addition, these approaches may potentially lower course budgets and participation fees, reducing economic barriers to course participation.

### 3.2 Online open access resources

Traditional “in‐person” material used in field courses could be developed into online open access resources, having the added value of facilitating sharing of knowledge to the broader ecological community. Linking into the Open Science framework, examples of open educational resources include freely accessible online lectures, tutorials or literature (Caswell et al., 2008; Hylen, 2006; McGreal et al., 2013). Practical components, such as online tutorials or workshops on how to design field campaigns, collect data, manage datasets and create a reproducible workflow, may also be made available (Fawcett, 2018; Kramer et al., 2018). For example, R tutorials based on data from previous course iterations could be offered as an online training which would also familiarize ECRs with established practices and formats.

### 3.3 Building local and global research networks

Online tools can play an important role in helping develop a sense of community and reciprocal responsibilities between ECRs during precourse activities that flows into the in‐person portion of the field course. Designing precourse activities to include collaborative coursework and providing platforms and opportunities for virtual “face‐to‐face” meetings and document development is critical for team building and collaboration (Lewis & Abdul‐Hamid, 2006). Online “break‐out rooms” can be used to support team‐based for example paper reviews or project development, with group learning known to increase performance outcomes (Springer et al., 1999). These “live” sessions can be supported by communication platforms, where conversations and documents can be easily shared. Online communication and collaboration platforms enhance group cohesion and facilitate productive postcourse online activities, including possible follow‐up projects and publications (Mansor, 2012). This approach develops a collaborative field course environment, as opposed to a hierarchical student/teacher set up, by allowing students to play an active role in constructing their learning experience (Carwile, 2007). Finally, linking to the broader research community, course participants and facilitators can use online platforms (i.e., websites, social media accounts, blogs) to promote educational and scientific practice (Sugimoto et al., 2017), as well as the ECRs themselves, increasing their scientific visibility at a critical stage in their career (Bielczyk et al., 2020). Ecological and evolutionary research is strengthened by global networks, which are often developed during early career attendance of field courses, workshops, and conferences. If done right, online components can significantly enhance these benefits, for instance by enabling professional as well as personal interaction long beyond course duration and in facilitating subsequent collaborations.

### 3.4 Acknowledging challenges in moving online

Lastly, we recognize that adopting online workflows for communication and collaboration in field courses may create new challenges (Berente & Howison, 2019). It is important that those designing platforms and materials for Open Science, as well as for specific courses, consider the accessibility, effectiveness, and engaging nature of the online tools or resources developed. Pedagogical discussions around online course delivery have been covered in depth (Dell et al., 2015; Schell & Janicki, 2013), so here we emphasize how effective remote teamwork requires good communication tools, trust, and compassion toward all team members. When integrating online approaches this is accomplished by ensuring accessibility of course content for all participants, including: considering Internet connectivity by reducing file sizes (Andersson, 2008); limiting the use of proprietary software; and anticipating and accommodating the diverse nature of participants, for example, by integrating “text to speech” and subtitle functionality (Dell et al., 2015). Successful courses require clear communication channels between course participants. When moving online this can be accomplished by: selecting appropriate online communication platforms—those that mimic a social media‐like experience have been shown to be effective (Sabin & Olive, 2018; Sclater et al., 2001); using video and audio sources to mimic the “face‐to‐face” interactions found in traditional courses to promote participant interaction and involvement (Lewis & Abdul‐Hamid, 2006) and therefore productivity (Kirschman & Greenstein, 2002); and being mindful of differences in working hours, that is, career responsibilities, commuting logistics, and time zones, when choosing meeting times (Tang et al., 2011), and possibly structuring subgroups based on location to ensure a degree of overlap (Heisman, 2020). Lastly, to assess the efficacy of not only the online components but of the course delivery as a whole, feedback from participants can be useful in identifying potential oversights which, in turn, can be used to adapt future course iterations accordingly.

## 4 CONCLUSION

The next generation of researchers is likely to work in a world where Open Science principles and workflows are increasingly mainstream and expected, not only within the research community, but also by funders, publishers, and society at large. To best equip them with the skills and competences they need to succeed in the future, we suggest field courses present an ideal platform to introduce Open Science practices. In the fields of ecology and evolution, we already have a strong scaffold of Open Science including registered reports, data documentation, standardized research protocols, and reproducible workflows; this scaffold aligns well with the structure of traditional field courses. In addition, combining online and field‐based learning following Open Science principles offers many possibilities to stimulate development of field courses, increase data and knowledge yield, and support ECR development. If chosen accordingly, online resources and activities can selectively supplement field‐based education to improve effectiveness of course time, widen educational impact, and facilitate networks among and beyond participants. Integrating these elements into field courses will benefit both ECRs and their mentors, course developers, and ultimately increase knowledge and research compatibility across the wider ecological community.

## CONFLICT OF INTEREST

None declared.

## AUTHOR CONTRIBUTIONS

**Sonya R. Geange:** Conceptualization (lead); visualization (equal); writing–original draft (lead); writing–review and editing (lead). **Jonathan von Oppen:** Conceptualization (lead); visualization (equal); writing–original draft (lead); writing–review and editing (lead). **Tanya Strydom:** Conceptualization (lead); visualization (lead); writing–original draft (equal); writing–review and editing (lead). **Mickey Boakye:** Conceptualization (lead); visualization (equal); writing–original draft (equal); writing–review and editing (equal). **Tasha‐Leigh J. Gauthier:** Conceptualization (lead); visualization (equal); writing–original draft (equal); writing–review and editing (lead). **Ragnhild Gya:** Conceptualization (lead); visualization (equal); writing–original draft (equal); writing–review and editing (equal). **Aud H. Halbritter:** Conceptualization (lead); visualization (equal); writing–original draft (equal); writing–review and editing (lead). **Laura H. Jessup:** Conceptualization (lead); visualization (equal); writing–original draft (equal); writing–review and editing (lead). **Sara L. Middleton:** Conceptualization (lead); visualization (equal); writing–original draft (equal); writing–review and editing (equal). **Jocelyn Navarro:** Conceptualization (lead); visualization (equal); writing–original draft (equal); writing–review and editing (equal). **Maria Elisa Pierfederici:** Conceptualization (lead); visualization (equal); writing–original draft (equal); writing–review and editing (equal). **Julia Chacon‐Labella:** Project administration (equal). **Sehoya Cotner:** Conceptualization (equal); funding acquisition (supporting); project administration (equal); writing–original draft (supporting); writing–review and editing (supporting). **William Farfan‐Rios:** Project administration (equal). **Brian S. Maitner:** Project administration (equal); writing–review and editing (supporting). **Sean T. Michaletz:** Project administration (equal). **Richard J. Telford:** Project administration (equal); Writing–review and editing (supporting). **Brian J. Enquist:** Funding acquisition (supporting); project administration (lead); writing–review and editing (supporting). **Vigdis Vandvik:** Conceptualization (equal); funding acquisition (lead); project administration (lead); visualization (equal); writing–original draft (equal); writing–review and editing (equal).

## Data Availability

Not applicable.

## References

[ece37009-bib-0001] Allen, C. , & Mehler, D. M. A. (2019). Open science challenges, benefits and tips in early career and beyond. PLoS Biology, 17(5), e3000246. 10.1371/journal.pbio.3000246 31042704 PMC6513108

[ece37009-bib-0002] Allen, I. E. , & Seaman, J. (2011). Going the distance: Online education in the United States.

[ece37009-bib-0003] Alston, J. , & Rick, J. (2020). A beginner’s guide to conducting reproducible research in ecology, evolution, and conservation. EcoEvoRxiv. 10.32942/osf.io/h5r6n

[ece37009-bib-0004] Andersson, A. (2008). Seven major challenges for e‐learning in developing countries: Case study eBIT, Sri Lanka. International Journal of Education and Development Using ICT, 4(3), 45–62.

[ece37009-bib-0005] Barrows, S. (1986). A taxonomy of problem‐based learning methods. Medical Education, 20, 481–486. 10.1111/j.1365-2923.1986.tb01386.x 3796328

[ece37009-bib-0006] Barton, C. , Smith, R. I. , & Weaver, R. (2010). Data practices, policy, and rewards in the information era demand a new paradigm. Data Science Journal, 9, IGY95–IGY99. 10.2481/dsj.SS_IGY-003

[ece37009-bib-0007] Barton, D. C. (2020). Impacts of the COVID‐19 pandemic on field instruction and remote teaching alternatives: Results from a survey of instructors. Ecology and Evolution, 1–9. 10.1002/ece3.6628 PMC7436523 32837715

[ece37009-bib-0008] Beltran, R. S. , Marnocha, E. , Race, A. , Croll, D. A. , Dayton, G. H. , & Zavaleta, E. S. (2020). Field courses narrow demographic achievement gaps in ecology and evolutionary biology. Ecology and Evolution, 10(12), 5184–5196. 10.1002/ece3.6300 32607142 PMC7319162

[ece37009-bib-0009] Berente, N. , & Howison, J. (2019). Strategies for success in virtual collaboration: Structures and norms for meetings, workflow, and technological platforms. In K. L. Hall , A. L. Vogel , & R. T. Croyle (Eds.), Strategies for team science success: Handbook of evidence‐based principles for cross‐disciplinary science and practical lessons learned from health researchers (pp. 563–574). Springer International Publishing.

[ece37009-bib-0010] Bielczyk, N. Z. , Ando, A. , Badhwar, A. , Caldinelli, C. , Gao, M. , Haugg, A. , Hernandez, L. M. , Ito, K. L. , Kessler, D. , Lurie, D. , Makary, M. M. , Nikolaidis, A. , Veldsman, M. , Allen, C. , Bankston, A. , Bottenhorn, K. L. , Braukmann, R. , Calhoun, V. , Cheplygina, V. , … Zhou, X. (2020). Effective self‐management for early career researchers in the natural and life sciences. Neuron, 106(2), 212–217. 10.1016/j.neuron.2020.03.015 32325057 PMC7665085

[ece37009-bib-0011] Biggs, J. (1996). Enhancing teaching through constructive alignment. Higher Education, 32(3), 347–364. 10.1007/BF00138871

[ece37009-bib-0012] Biggs, J. B. , & Tang, C. (2011). Teaching for quality learning at university. McGraw‐Hill/Society for Research into Higher Education/Open University Press.

[ece37009-bib-0013] Borer, E. T. , Harpole, W. S. , Adler, P. B. , Lind, E. M. , Orrock, J. L. , Seabloom, E. W. , Smith, M. D. , & Freckleton, R. (2014). Finding generality in ecology: A model for globally distributed experiments. Methods in Ecology and Evolution, 5(1), 65–73. 10.1111/2041-210x.12125

[ece37009-bib-0014] Borer, E. T. , Seabloom, E. W. , Jones, M. B. , & Schildhauer, M. (2009). Some simple guidelines for effective data management. The Bulletin of the Ecological Society of America, 90(2), 205–214. 10.1890/0012-9623-90.2.205

[ece37009-bib-0015] Borregaard, M. K. , & Hart, E. M. (2016). Towards a more reproducible ecology. Ecography, 39(4), 349–353. 10.1111/ecog.02493

[ece37009-bib-0016] Carwile, J. (2007). A constructivist approach to online teaching and learning. Inquiry, 12(1), 68–73.

[ece37009-bib-0017] Caswell, T. , Henson, S. , Jensen, M. , & Wiley, D. (2008). Open content and open educational resources: Enabling universal education. The International Review of Research in Open and Distributed Learning, 9(1), 1–11. 10.19173/irrodl.v9i1.469

[ece37009-bib-0018] Chambers, C. (2019). What’s next for Registered Reports? Nature, 573(7773), 187–189. 10.1038/d41586-019-02674-6 31506624

[ece37009-bib-0019] Christensen, G. , Steinmetz, A. , Alcorn, B. , Bennett, A. , Woods, D. , & Emanuel, E. J. (2013). The MOOC phenomenon: Who takes massive open online courses and why? SSRN Electronic Journal. 10.2139/ssrn.2350964

[ece37009-bib-0020] Coalition‐S , (2020). Plan S’ and ‘cOAlition S’ – Accelerating the Transition to Full and Immediate Open Access to Scientific Publications. accessed July 2, 2020. Retrieved from https://www.coalition‐s.org/

[ece37009-bib-0021] Cook, R. B. , Olson, R. J. , Kanciruk, P. , & Hook, L. A. (2001). Best practices for preparing ecological data sets to share and archive. Bulletin of the Ecological Society of America, 82(2), 138–141.

[ece37009-bib-0022] Cotner, S. , Enquist, B. J. , Chacon, J. , Maitner, B. S. , Farfan‐Rios, W. , Michaletz, S. T. , Garen, J. , Gauthier, T. , Vandvik, V. , Gya, R. , Halbritter, A. H. , Gaudard, J. , Hoskova, K. , Pierfederici, M. , Quinteros‐Casaverde, N. , Sanchez‐Diaz, E. , Jessup, L. , Strydom, T. , & Von Oppen, J. (2020). International scientists need better support during global emergencies. Retrieved from https://www.timeshighereducation.com/blog/international‐scientists‐need‐better‐support‐during‐global‐emergencies

[ece37009-bib-0023] Cousijn, H. , Feeney, P. , Lowenberg, D. , Presani, E. , & Simons, N. (2019). Bringing citations and usage metrics together to make data count. Data Science Journal, 18, 9. 10.5334/dsj-2019-009

[ece37009-bib-0024] D'Amato, L. G. , & Krasny, M. E. (2011). Outdoor adventure education: Applying transformative learning theory to understanding instrumental learning and personal growth in environmental education. The Journal of Environmental Education, 42(4), 237–254. 10.1080/00958964.2011.581313

[ece37009-bib-0025] Dell, C. A. , Dell, T. F. , & Blackwell, T. L. (2015). Applying universal design for learning in online courses: Pedagogical and practical considerations. Journal of Educators Online, 12(2), 166–192. 10.9743/JEO.2015.2.1

[ece37009-bib-0026] Durrant, K. L. , & Hartman, T. P. V. (2015). The integrative learning value of field courses. Journal of Biological Education, 49(4), 385–400. 10.1080/00219266.2014.967276

[ece37009-bib-0027] European Commission (2018). Turning FAIR into reality: Final report and action plan from the European Commission Expert Group on FAIR Data. Publications Office of the European Union.

[ece37009-bib-0028] European Commission (2020). Progress on open science: Towards a shared research knowledge system – Final report of the open science policy platform. Directorate‐General for Research and Innovation.

[ece37009-bib-0029] Ewers, R. M. , Barlow, J. , Banks‐Leite, C. , & Rahbek, C. (2019). Separate authorship categories to recognize data collectors and code developers. Nature Ecology & Evolution, 3(12), 1610. 10.1038/s41559-019-1033-9 31686016

[ece37009-bib-0030] Farnham, A. , Kurz, C. , Öztürk, M. A. , Solbiati, M. , Myllyntaus, O. , Meekes, J. , Pham, T. M. , Paz, C. , Langiewicz, M. , Andrews, S. , Kanninen, L. , Agbemabiese, C. , Guler, A. T. , Durieux, J. , Jasim, S. , Viessmann, O. , Frattini, S. , Yembergenova, D. , Benito, C. M. , … Hettne, K. (2017). Early career researchers want Open Science. Genome Biology, 18(1), 221. 10.1186/s13059-017-1351-7 29141654 PMC5688730

[ece37009-bib-0031] Fawcett, L. (2018). Using interactive shiny applications to facilitate research‐informed learning and teaching. Journal of Statistics Education, 26(1), 2–16. 10.1080/10691898.2018.1436999

[ece37009-bib-0032] Fecher, B. , & Friesike, S. (2013). Open science: One term, five schools of thought. Opening Science, 17–47.

[ece37009-bib-0033] Feng, X. , Qiao, H. , & Enquist, B. J. (2020). Doubling demands in programming skills call for ecoinformatics education. Frontiers in Ecology and the Environment, 18(3), 123–124. 10.1002/fee.2179

[ece37009-bib-0034] Fleischner, T. L. , Espinoza, R. E. , Gerrish, G. A. , Greene, H. W. , Kimmerer, R. W. , Lacey, E. A. , Pace, S. , Parrish, J. K. , Swain, H. M. , Trombulak, S. C. , Weisberg, S. , Winkler, D. W. , & Zander, L. (2017). Teaching biology in the field: Importance, challenges, and solutions. BioScience, 67(6), 558–567. 10.1093/biosci/bix036

[ece37009-bib-0035] Foster, E. , & Deardorff, A. (2017). Open Science Framework (OSF). Journal of the Medical Library Association, 105(2), 203. 10.5195/JMLA.2017.88

[ece37009-bib-0036] Fraser, L. H. , Henry, H. A. , Carlyle, C. N. , White, S. R. , Beierkuhnlein, C. , Cahill, J. F. Jr , Casper, B. B. , Cleland, E. , Collins, S. L. , Dukes, J. S. , & Knapp, A. K. (2013). Coordinated distributed experiments: an emerging tool for testing global hypotheses in ecology and environmental science. Frontiers in Ecology and the Environment, 11(3), 147–155. 10.1890/110279

[ece37009-bib-0037] Gallagher, R. V. , Falster, D. S. , Maitner, B. S. , Salguero‐Gómez, R. , Vandvik, V. , Pearse, W. D. , Schneider, F. D. , Kattge, J. , Poelen, J. H. , Madin, J. S. , Ankenbrand, M. J. , Penone, C. , Feng, X. , Adams, V. M. , Alroy, J. , Andrew, S. C. , Balk, M. A. , Bland, L. M. , Boyle, B. L. , … Enquist, B. J. (2020). Open Science principles for accelerating trait‐based science across the Tree of Life. Nature Ecology & Evolution, 4, 294–303. 10.1038/s41559-020-1109-6 32066887

[ece37009-bib-0038] Goodenough, A. E. , Rolfe, R. N. , MacTavish, L. , & Hart, A. G. (2014). The role of overseas field courses in student learning in the biosciences. Bioscience Education, 1–15. 10.11120/beej.2014.00021

[ece37009-bib-0039] Gore, H. (2014). Massive Open Online Courses (MOOCs) and their impact on academic library services: Exploring the issues and challenges. New Review of Academic Librarianship, 20(1), 4–28. 10.1080/13614533.2013.851609

[ece37009-bib-0040] Grainger, M. J. , Bolam, F. C. , Stewart, G. B. , & Nilsen, E. B. (2020). Evidence synthesis for tackling research waste. Nature Ecology & Evolution, 4(4), 495–497. 10.1038/s41559-020-1141-6 32203478

[ece37009-bib-0041] Gurevitch, J. , Curtis, P. S. , & Jones, M. H. (2001). 'Meta‐analysis in ecology' in Advances in Ecological Research (pp. 199–247). Academic Press.

[ece37009-bib-0042] Haddaway, N. R. (2018). Open synthesis: On the need for evidence synthesis to embrace Open Science. Environmental Evidence, 7(1), 1–5. 10.1186/s13750-018-0140-4

[ece37009-bib-0043] Haddaway, N. R. , & Verhoeven, J. T. A. (2015). Poor methodological detail precludes experimental repeatability and hampers synthesis in ecology. Ecology and Evolution, 5(19), 4451–4454. 10.1002/ece3.1722 26664691 PMC4667817

[ece37009-bib-0044] Halbritter, A. H. , DeBoeck, H. J. , Eycott, A. E. , Reinsch, S. , Robinson, D. A. , Vicca, S. , Berauer, B. , Christiansen, C. T. , Estiarte, M. , Grünzweig, J. M. , Gya, R. , Hansen, K. , Jentsch, A. , Lee, H. , Linder, S. , Marshall, J. , Peñuelas, J. , Kappel Schmidt, I. , Stuart‐Haëntjens, E. , … Zurba, K. (2019) The handbook for standardized field and laboratory measurements in terrestrial climate change experiments and observational studies (ClimEx). Methods in Ecology and Evolution, 11(1), 22–37. 10.1111/2041-210X.13331

[ece37009-bib-0045] Hampton, S. E. , Anderson, S. S. , Bagby, S. C. , Gries, C. , Han, X. , Hart, E. M. , Jones, M. B. , Lenhardt, W. C. , MacDonald, A. , Michener, W. K. , Mudge, J. , Pourmokhtarian, A. , Schildhauer, M. P. , Woo, K. H. , & Zimmerman, N. (2015). The Tao of open science for ecology. Ecosphere, 6(7), art120. 10.1890/es14-00402.1

[ece37009-bib-0046] Heisman, L. (2020). Remote work: Sharing tips for leading in a remote world. accessed 07/09/2020 Retrieved from https://github.blog/2020‐08‐07‐remote‐work‐sharing‐tips‐for‐leading‐in‐a‐remote‐world/

[ece37009-bib-0047] Henry, G. H. R. , & Molau, U. (1997). Tundra plants and climate change: The International Tundra Experiment (ITEX). Global Change Biology, 3(S1), 1–9. 10.1111/j.1365-2486.1997.gcb132.x

[ece37009-bib-0048] Hines, S. L. , Vedral, A. J. , Jefferson, A. E. , Drymon, J. M. , Woodrey, M. S. , Mabey, S. E. , & Sparks, E. L. (2020). Engaging online students by activating ecological knowledge. Ecology and Evolution, 1–10. 10.1002/ece3.6739 PMC7679540 33250987

[ece37009-bib-0049] Hole, T. N. (2018). Working and learning in a field excursion. Cbe—life Sciences Education, 17(2), ar24. 10.1187/cbe.17-08-0185 29749842 PMC5998312

[ece37009-bib-0050] Hylen, J. (2006). Open educational resources: Opportunities and challenges. In Proceedings of Open Education.

[ece37009-bib-0051] Inouye, D. W. , Underwood, N. , Inouye, B. D. , & Irwin, R. E. (2020). Support early‐career field researchers. Science, 368(6492), 724.2–725. 10.1126/science.abc1261 32409467

[ece37009-bib-0052] Kattge, J. , Boenisch, G. , Diaz, S. , Lavorel, S. , Prentice, C. , Leadley, P. , Wirth, C. , & The T. R. Y. Consortium (2020). The TRY Plant Trait Database ‐ Enhanced coverage and open access. Global Change Biology, 26(1), 119–199. 10.5194/egusphere-egu2020-20191 31891233

[ece37009-bib-0053] Kervinen, A. , Uitto, A. , & Juuti, K. (2018). How fieldwork‐oriented biology teachers establish formal outdoor education practices. Journal of Biological Education, 54(2), 115–128. 10.1080/00219266.2018.1546762

[ece37009-bib-0054] Kirschman, J. S. , & Greenstein, J. S. (2002). The use of groupware for collaboration in distributed student engineering design teams. Journal of Engineering Education, 91(4), 403–407. 10.1002/j.2168-9830.2002.tb00724.x

[ece37009-bib-0055] Klingberg, T. (2012). Slik lærer hjernen: hvordan barn husker og lærer.

[ece37009-bib-0056] Kramer, M. , Olson, D. , & Walker, J. (2018). Design and assessment of online, interactive tutorials that teach science process skills. Cbe—life Sciences Education, 17(2), ar19. 10.1187/cbe.17-06-0109 29749846 PMC5998323

[ece37009-bib-0057] Kricsfalusy, V. , George, C. , & Reed, M. G. (2018). Integrating problem‐ and project‐based learning opportunities: Assessing outcomes of a field course in environment and sustainability. Environmental Education Research, 24(4), 593–610. 10.1080/13504622.2016.1269874

[ece37009-bib-0058] Lannom, L. , Koureas, D. , & Hardisty, A. R. (2020). FAIR data and services in biodiversity science and geoscience. Data Intelligence, 2(1–2), 122–130. 10.1162/dint_a_00034

[ece37009-bib-0059] Lembrechts, J. J. , Aalto, J. , Ashcroft, M. B. , De Frenne, P. , Kopecky, M. , Lenoir, J. , Luoto, M. , Maclean, I. M. D. , Roupsard, O. , Fuentes‐Lillo, E. , Garcia, R. A. , Pellissier, L. , Pitteloud, C. , Alatalo, J. M. , Smith, S. W. , Bjork, R. G. , Muffler, L. , Ratier Backes, A. , Cesarz, S. , … Nijs, I. (2020). SoilTemp: A global database of near‐surface temperature. Global Change Biology, 26(11), 6616–6629. 10.1111/gcb.15123 32311220

[ece37009-bib-0060] Leon‐Beck, M. , & Dodick, J. (2012). Exposing the challenges and coping strategies of field‐ecology graduate students. International Journal of Science Education, 34(16), 2455–2481. 10.1080/09500693.2012.713145

[ece37009-bib-0061] Lewis, C. C. , & Abdul‐Hamid, H. (2006). Implementing effective online teaching practices: Voices of exemplary faculty. Innovative Higher Education, 31(2), 83–98. 10.1007/s10755-006-9010-z

[ece37009-bib-0062] Lowndes, J. S. S. , Best, B. D. , Scarborough, C. , Afflerbach, J. C. , Frazier, M. R. , O’Hara, C. C. , Jiang, N. , & Halpern, B. S. (2017). Our path to better science in less time using open data science tools. Nature Ecology & Evolution, 1(6), 160, 10.1038/s41559-017-0160 28812630

[ece37009-bib-0063] Luc, J. G. Y. , Archer, M. A. , Arora, R. C. , Bender, E. M. , Blitz, A. , Cooke, D. T. , Hlci, T. N. , Kidane, B. , Ouzounian, M. , Varghese, T. K. , & Antonoff, M. B. (2020). Does tweeting improve citations? One‐year results from the TSSMN Prospective Randomized Trial. The Annals of Thoracic Surgery. 10.1016/j.athoracsur.2020.04.065 32504611

[ece37009-bib-0064] Mansor, A. Z. (2012). Google docs as a collaborating tool for academicians. Procedia ‐ Social and Behavioral Sciences, 59, 411–419. 10.1016/j.sbspro.2012.09.295

[ece37009-bib-0065] McGreal, R. , Kinuthia, W. , & Marshall, S. (2013). Open educational resources: Innovation, research and practice. Vancouver.

[ece37009-bib-0066] Michener, W. K. (2006). Meta‐information concepts for ecological data management. Ecological Informatics, 1(1), 3–7. 10.1016/j.ecoinf.2005.08.004

[ece37009-bib-0067] Mislan, K. A. S. , Heer, J. M. , & White, E. P. (2016). Elevating the status of code in ecology. Trends in Ecology & Evolution, 31(1), 4–7. 10.1016/j.tree.2015.11.006 26704455

[ece37009-bib-0068] Mongeon, P. , Robinson‐Garcia, N. , Jeng, W. , & Costas, R. (2017). Incorporating data sharing to the reward system of science. Aslib Journal of Information Management, 69(5), 545–556. 10.1108/ajim-01-2017-0024

[ece37009-bib-0069] Nakagawa, S. , Dunn, A. G. , Lagisz, M. , Bannach‐Brown, A. , Grames, E. M. , Sánchez‐Tójar, A. , O’Dea, R. E. , Noble, D. W. A. , Westgate, M. J. , Arnold, P. A. , Barrow, S. , Bethel, A. , Cooper, E. , Foo, Y. Z. , Geange, S. R. , Hennessy, E. , Mapanga, W. , Mengersen, K. , Munera, C. , … Haddaway, N. R. (2020). A new ecosystem for evidence synthesis. Nature Ecology & Evolution, 4(4), 498–501. 10.1038/s41559-020-1153-2 32203483

[ece37009-bib-0070] Park, D. S. (2020). The invisible university is COVID‐19 positive. Trends in Genetics, 36(8), 543–544. 10.1016/j.tig.2020.05.010 32518044 PMC7253946

[ece37009-bib-0071] Parker, T. H. , Forstmeier, W. , Koricheva, J. , Fidler, F. , Hadfield, J. D. , Chee, Y. E. , Kelly, C. D. , Gurevitch, J. , & Nakagawa, S. (2016). Transparency in ecology and evolution: real problems, real solutions. Trends in Ecology & Evolution, 31(9), 711–719. 10.1016/j.tree.2016.07.002 27461041

[ece37009-bib-0072] Peasland, E. L. , Henri, D. C. , Morrell, L. J. , & Scott, G. W. (2019). The influence of fieldwork design on student perceptions of skills development during field courses. International Journal of Science Education, 41(17), 2369–2388. 10.1080/09500693.2019.1679906

[ece37009-bib-0073] Perez‐Harguindeguy, N. , Diaz, S. , Garnier, E. , Lavorel, S. , Poorter, H. , Jaureguiberry, P. , Bret‐Harte, M. S. , Cornwell, W. K. , Craine, J. M. , Gurvich, D. E. , Urcelay, C. , Veneklaas, E. J. , Reich, P. B. , Poorter, L. , Wright, I. J. , Ray, P. , Enrico, L. , Pausas, J. G. , de Vos, A. C. , … Cornelissen, J. H. C. . (2013). New handbook for standardised measurement of plant functional traits worldwide. Australian Journal of Botany, 61(3), 167–234. 10.1071/BT12225

[ece37009-bib-0074] Powers, S. M. , & Hampton, S. E. (2019). Open science, reproducibility, and transparency in ecology. Ecological Applications, 29(1), e01822, 10.1002/eap.1822 30362295

[ece37009-bib-0075] Sabin, J. , & Olive, A. (2018). Slack: Adopting social‐networking platforms for active learning. PS: Political Science & Politics, 51(1), 183–189.

[ece37009-bib-0076] Savery, J. R. (2015). Overview of problem‐based learning: Definitions and distinctions. Essential Readings in problem‐based Learning: Exploring and Extending the Legacy of Howard S. Barrows, 9, 5–15.

[ece37009-bib-0077] Schell, G. , & Janicki, T. J. (2013). Online course pedagogy and the constructivist learning model. Journal of the Southern Association for Information Systems, 1(1), 26–36. 10.3998/jsais.11880084.0001.104

[ece37009-bib-0078] Sclater, N. , Grierson, H. , Ion, W. J. , & MacGregor, S. P. (2001). Online collaborative design projects: Overcoming barriers to communication. International Journal of Engineering Education, 17(2), 189–196.

[ece37009-bib-0079] Springer, L. , Stanne, M. E. , & Donovan, S. S. (1999). Effects of small‐group learning on undergraduates in science, mathematics, engineering, and technology: A meta‐analysis. Review of Educational Research, 69(1), 21–51. 10.3102/00346543069001021

[ece37009-bib-0080] Strasser, C. R. , Cook, R. , Michener, W. K. , & Budden, A. (2018). Primer on data management: What you always wanted to know. Retrieved from https://www.dataone.org/sites/all/documents/DataONEBPPrimer020212.pdf

[ece37009-bib-0081] Sugimoto, C. R. , Work, S. , Larivière, V. , & Haustein, S. (2017). Scholarly use of social media and altmetrics: A review of the literature. Journal of the Association for Information Science and Technology, 68(9), 2037–2062. 10.1002/asi.23833

[ece37009-bib-0082] Tang, J. C. , Zhao, C. , Cao, X. , & Inkpen, K. (2011). Your time zone or mine? A study of globally time zone‐shifted collaboration. In Proceedings of the ACM 2011 conference on Computer supported cooperative work (pp. 235–244). Association for Computing Machinery. 10.1145/1958824.1958860

[ece37009-bib-0083] Tennant, J. , Beamer, J. , Bosman, J. , Brembs, B. , Chung, N. C. , Clement, G. , Crick, T. , Dugan, J. , Dunning, A. , Eccles, D. , Enkhbayar, A. , Graziotin, D. , Harding, R. , Havemann, J. , Katz, D. , Khanal, K. , Norgaard, K. , Koder, T. , Macklin, P. , … Tuner, A. (2019). Foundations for open scholarship strategy development. MetaArXiv Preprints. 10.31222/osf.io/b4v8p

[ece37009-bib-0084] Toelch, U. , & Ostwald, D. (2018). Digital open science—Teaching digital tools for reproducible and transparent research. PLoS Biology, 16(7), e2006022. 10.1371/journal.pbio.2006022 30048447 PMC6095603

[ece37009-bib-0085] Van Noorden, R. (2014). Online collaboration: Scientists and the social network. Nature, 512(7513), 126–129. 10.1038/512126a 25119221

[ece37009-bib-0086] Vandvik, V. , Halbritter, A. H. , Yang, Y. , He, H. , Zhang, L. , Brummer, A. B. , Klanderud, K. , Maitner, B. S. , Michaletz, S. T. , Sun, X. , Telford, R. J. , Wang, G. , Althuizen, I. H. J. , Henn, J. J. , Garcia, W. F. E. , Gya, R. , Jaroszynska, F. , Joyce, B. L. , Lehman, R. , … Enquist, B. J. (2020). Plant traits and vegetation data from climate warming experiments along an 1100 m elevation gradient in Gongga Mountains, China. Scientific Data, 7(1), 189. 10.1038/s41597-020-0529-0 32561854 PMC7305152

[ece37009-bib-0087] Vuorre, M. , & Curley, J. P. (2018). Curating research assets: A tutorial on the Git version control system. Advances in Methods and Practices Psychological Science, 1(2), 219–236. 10.1177/2515245918754826

[ece37009-bib-0088] Wieczorek, J. , Bloom, D. , Guralnick, R. , Blum, S. , Döring, M. , Giovanni, R. , Robertson, T. , & Vieglais, D. (2012). Darwin core: An evolving community‐developed biodiversity data standard. PLoS One, 7(1), e29715. 10.1371/journal.pone.0029715 22238640 PMC3253084

[ece37009-bib-0089] Wilkinson, M. D. , Dumontier, M. , Aalbersberg, I. J. , Appleton, G. , Axton, M. , Baak, A. , Blomberg, N. , Boiten, J.‐W. , da Silva Santos, L. B. , Bourne, P. E. , Bouwman, J. , Brookes, A. J. , Clark, T. , Crosas, M. , Dillo, I. , Dumon, O. , Edmunds, S. , Evelo, C. T. , … Mons, B. (2016) The FAIR Guiding Principles for scientific data management and stewardship. Scientific Data, 3(1), 1–9. 10.1038/sdata.2016.1 PMC4792175 26978244

[ece37009-bib-0090] Wolkovich, E. M. , Regetz, J. , & O'Connor, M. I. (2012). Advances in global change research require open science by individual researchers. Global Change Biology, 18(7), 2102–2110. 10.1111/j.1365-2486.2012.02693.x

